# Male Pronuclear Formation and Embryo Development Following Intracytoplasmic Injection of Ovine Pretreated Sperm

**Published:** 2018

**Authors:** Abolfazl Shirazi, Arefeh Golestanfar, Masomeh Bashiri, Ebrahim Ahmadi, Naser Shams-Esfandabadi

**Affiliations:** 1. Reproductive Biotechnology Research Center, Avicenna Research Institute, ACECR, Tehran, Iran; 2. Department of Gametes and Cloning, Research Institute of Animal Embryo Technology, Shahrekord University, Shahrekord, Iran

**Keywords:** Intracytoplasmic sperm injection (ICSI), Male, Ovine, Pronucleus

## Abstract

**Background:**

Failure of Male Pronucleus (MPN) formation is a major concern in the success of Intracytoplasmic Sperm Injection (ICSI) in some species. Despite the conducted unsuccessful efforts to improve ICSI efficiency in ovine, the present study was aimed to improve MPN formation and embryo development in ovine ICSI procedure through accompaniment of sperm pretreatment with co-injection of some compounds.

**Methods:**

In experiment 1, sperm were treated with either 2-mercaptoethanol (2ME), glutathione (GSH), or DTT (dithiothreitol) in combination with Heparin (Hep). Following DNA integrity and fragmentation assessments, the best sperm pretreatment approach in induction of sperm head decondensation was applied for the second and third experiments. In experiment 2, *in vitro* matured oocytes were subjected to ICSI using pretreated sperm with or without GSH and Sperm Extract (SE) co-injection. In experiment 3, the procedure was followed as experiment 2 with acrosome reacted sperm.

**Results:**

The highest percentages of oocyte activation were observed in Hep+GSH and Hep+2ME groups. The greatest MPN formations were also observed in the same groups when ICSI procedure was accompanied with GSH co-injection. Despite the higher percentage of MPN formation and oocyte activation in Hep+GSH and Hep+2ME groups, none of the employed strategies could increase the cleavage and blastocyst rates compared to the control.

**Conclusion:**

In our study condition, despite the lack of significant increase in embryo development in treated groups, the significant increase in MPN formation in Hep+ GSH+co.GSH and Hep+2ME+co.GSH groups indicates the lower chance of parthenote formation that means a higher chance of normal fertilization compared with control.

## Introduction

Intracytoplasmic Sperm Injection (ICSI) as an integral part of assisted reproduction has become increasingly popular in human male patients with certain infertility problems ^1^. In animal species, this technique has several applications such as avoidance of the polyspermy problem (*e.g*. porcine) ^2^, extending the sperm vector system for transgenic animal production, and preserving the endangered species. ICSI also provides an opportunity for research into cell cycle control and mechanisms involved in sperm-induced oocyte activation ^3^.

A problem commonly encountered in ICSI technique is the low rate of viability of the microinjected oocytes and inconsistent level of male pronuclear formation after microinjection. The inadequate sperm chromatin decondensation and its transformation into the Male Pronucleus (MPN) together with a failure to activate the oocyte seem to be the major causes behind the poor ICSI efficiency in some species ^4,5^.

In this context, while ICSI alone in some species such as mice, humans, hamsters, and rabbits is sufficient to activate the oocytes for further embryonic development ^6–10^, in other species such as cattle, sheep, pig ^11,12^, buffalo ^13^, and horse ^14,15^, an additional parthenogenetic activation is necessary to activate the oocytes after ICSI ^16^. The nuclei of mammalian spermatozoa are genetically inactivated and structurally stabilized by association of sperm DNA with protamines. Under *in vitro* conditions, decondensation of mammalian sperm nuclei can be induced by sperm pretreatment with a disulfide-reducing agent alone ^17^ or together with neutral detergents ^18^, anionic detergents ^19^, proteases ^20^, salts ^21^ or mechanical demembranation ^22^. In mice, the success of ICSI could be improved by the removal of both sperm membranes and acrosome before injection ^23^. Moreover, sperm pretreatment can affect the ability of oocytes to activate ^12^.

Among mechanical pre-treatments, sonication ^24^ and repetitive freezing/thawing without cryoprotectants ^25^ have been reported to improve MPN formation after ICSI. However, sperm pre-treatment with DTT ^26^ and Triton X-100 ^26^ or sperm freezing ^27^ has been documented to cause reduction of oocyte-activating capacity of porcine oocytes following ICSI. These treatments were aimed to remove sperm membrane so that to improve male pronuclear formation and to speed up the availability of sperm-borne oocyte activation factor to the oocyte ^5,28^.

Regarding the strategies applied on the sperm to improve normal fertilization and embryo development after ICSI, several treatment regimens have been tested to disrupt or even remove the sperm plasma membrane to allow a more direct interaction of sperm with the oocyte cytoplasm.

In line with our previous studies ^12^ indicating the low efficiency of ICSI in sheep, the purpose of this study was to evaluate the influence of several strategies to improve male pronuclear formation and normal fertilization following ICSI in this species.

## Materials and Methods

Except where otherwise indicated, all chemicals were obtained from the Sigma (St. Louis, MO, USA).

### Oocyte collection and in vitro maturation

Abattoir obtained sheep ovaries were transported to the laboratory in saline (25–30 °C) in a thermos flask within 2-3 *hr*. Ovaries were washed three times with pre-warmed fresh saline (37 °*C*), and all visible follicles with a diameter of 2–6 *mm* were aspirated using gentle vacuum (30 *mmHg*) *via* a 20 gauge short beveled needle connected to a vacuum pump. Prior to aspiration, the collecting tube was filled with 2 *ml* preincubated hepes-modified TCM, supplemented with 50 *IU/ml* heparin. After aspiration, only oocytes surrounded by more than three layers of unexpanded cumulus cells (COCs: cumulus oocyte complexes) were selected for *in vitro* maturation (IVM). Before culturing, oocytes were washed in Hepes-buffered TCM199 (HTCM199) supplemented with 5% FBS (Fetal Bovine Serum, Gibco 10270), and 2 *mM* glutamine. The oocyte culture medium (OCM) consisted of bicarbonate buffered TCM 199 with 2 *mM* L-glutamine supplemented with 0.02 *mg/ml* cysteamine, 1 *IU/ml* hCG, 0.1 *IU/ml* FSH, 100 *ml/ml* penicillin, 100 *mg/ml* streptomycin, 10% FBS (Fetal bovine serum, Gibco 10270), and 0.2 *mM* Na-Pyruvate. The medium osmolarity was adjusted to 275 *mOsm*. The oocytes were randomly distributed in maturation droplets (10 oocytes in 50 *μl*) and covered by sterile paraffin oil in a 60-*mm* Petri dish (Falcon 1008; Becton & Dickinson, Lincoln Park, NJ) and were then incubated under an atmosphere of 5% CO_2_ and 95% air with 100% humidity at 39 °*C* for 24 *hr*.

### Experimental Design

Experiment 1: The sperm were incubated in the presence of different concentrations of disulfide-reducing agents (2ME, GSH, and DTT) in combination with heparin at different incubation times. After incubation and following assessment of DNA fragmentation (Tunnel assay) and chromatin integrity (Acridine orange staining) of pretreated sperm, two of the best treatment regimens with appropriate sperm head decondensation (7 to 9 *μm*) were considered for the second and third experiments.
Experiment 2: The pretreated sperm was applied in ICSI procedure and the effect of GSH and Sperm Extract (SE) co-injection with pretreated sperm was also evaluated.
Experiment 3: The procedure was the same as second experiment except that the sperm acrosome already had been reacted with inomycine.

### Sperm preparation

The procedure was the same as previously described with minor modification ^29^. The 0.25 *ml* straw of frozen pooled semen was thawed at 37 °*C* in a water bath for 30 *s*. The same pooled semen was applied throughout the experiment. The thawed semen was then layered on top of two layers of Percoll density gradient consisting of 1 *ml* of each of 40% and 90% Percoll in a 15 *ml* conical centrifuge tube. The tube was then centrifuged at 650×*g* for 5 *min*, after which the supernatant was removed, leaving only the sperm pellet. The sperm pellet was washed at 600×*g* for 5 *min* using 1 *ml* Tyrode’s Albumin Lactate Pyruvate (TALP). The supernatant was removed leaving 100 *μl* containing the sperm suspension in the tube, which was then used for further treatment ^29^.

### In vitro sperm head decondensation (heparin plus 2ME, GSH, and DTT)

The sperm suspensions were incubated in the presence of the following chemicals: I) heparin (80 *μM*)+2-mercaptoethanol (2.5 and 5 *mM*) for 60, 90, 120, and 180 *min*; II) heparin (80 *μM*)+glutathione (15 *mM*) for 15, 30, 45, and 60 *min*; and III) heparin (80 *μM*)+ dithiothreitol (2.5 and 5 *mM*) for 60, 90, 120, and 180 *min* ^12,30,31^. The two more desirable regimens in sperm nuclear decondensation with the least DNA breakage or fragmentation (7 *μm* <sperm head expansion <9 *μm*) were considered for experiments 2 and 3.

### Spermatozoa DNA fragmentation

The prepared smeared sperm after 60 *min* fixation in 4% paraformaldehyde were permeabilized on ice with 0.1% (*v/v*) Triton X-100 in sodium citrate for 2 *min*. Detection of DNA fragmentation was performed using an In Situ Cell Death Detection Kit (Roche, Mannheim, Germany) according to the manufacturer’s instructions. Briefly, the slides were washed twice in PBS and incubated with the TUNEL reaction mixture for 1 *hr* at 37 °*C* in a humidified chamber. The sperm chromatins were counterstained using 5 *pg/ml* of Hoechst 33342. The TUNEL-negative sperm fluorescent red, while the TUNEL-positive (apoptotic) sperm fluoresced bright green (observing 200 sperm with high magnification/slide) ^32^.

### Acridine orange staining

Briefly, 30 *μl* of pretreated sperm suspension was smeared on slide and after being dried in air was fixed in the presence of Carnoy’s solution (methanol-acetic acid 3:1) for 2 *hr*. The slide was then stained with AO solution for 10 *min* at dark. The stained slides were rinsed with distilled water and after drying were inspected under fluorescent microscope for assessment of sperm head decondensation and chromatin integrity (observing 200 sperm with high magnification/slide). The sperm with intact and denatured (any breakage) DNA were stained as green and yellow-red colored, respectively ^33^.

### Induction of the acrosome reaction

The rich fraction of percoll isolated sperm was treated in H-TCM containing 2% serum supplemented with 10 *μM* ionomycin at 37 °*C* for 45 *min*. The solution was then centrifuged at 200 *g* for 1 *min* and the small droplet of sperm was subjected to gelatinolysis test to assess the acrosome status.

### Assessment of acrosome integrity (gelatinolysis test)

Gelatinolysis test was carried out according to Henkel *et al* ^34^ with minor modification. Twenty to 30 microliters of 5% gelatin solution were smeared on slide and after drying in air were placed at 4°*C* overnight. The slides were then fixed with 0.05% glutaraldehyde for 2–3 *min* and then thoroughly washed with distilled water. Twenty microliters of processed semen sample after dilution (1:10) in phosphate-buffered saline were smeared on precoated gelatin slides and incubated in a moist chamber at 37°*C* for 2 *hr*. The halo diameter around 200 spermatozoa using phase contrast microscope was evaluated and the percentage of spermatozoa showing a halo (sperm with intact acrosome) was calculated per slide.

### Preparation of sperm extract

Sperm extract was prepared based on Perry *et al* ^35^ protocol with a minor modification. Briefly, the frozen/thawed semen after dilution in Nuclear Isolation Medium (NIM: 125 *mM* KCl, 2.6 *mM* NaCl, 7.8 *mM* Na2HPO4, 1.4 *mM* KH2PO4, 3.0 *mM* EDTA; pH= 7.45) was pelleted after centrifugation for 3 *min* at 1500×*g*, three times, at room temperature. All subsequent steps were performed at 0–4 °*C*. The sperm pellet was resuspended in NIM (giving 2–10×107 *sperm/ml*) containing 0.05–0.1% (*v:v*) Triton X-100. The sperm suspension was subjected to three10-sec bursts of sonication (60% output, FAPAN ultrasound Prob-400R sonicator, Iran). Sperm fragments were pelleted for 2 *min* at 20,000×*g* and then washed twice thoroughly in NIM at 2 °*C*, with pelleting times of 6 and 25 *min* (20 000×*g*). The pellets were resuspended in 100 *ml* NIM (giving 2–10×107 *sperm/ml*) containing 15 *mM* dithiothreitol (DTT) and incubated for 30 *min* at 27 °*C* in order to dissolve SOAF located in sperm perinuclear theca. The sperm were then pelleted for 50–80 *min* spinning at 20,000×*g* at 2 °*C*. The cell-free supernatant produced from the suspensions was carefully removed and co-injected on the day of preparation.

### Intracytoplasmic sperm injection (ICSI)

ICSI was performed as previously described ^12^. Within 1 *hr* after injection, the oocytes were activated by exposure to 5 *mM* ionomycin in H-SOF containing 3% FBS for 5 *min* and then cultured in IVF-SOF for 3 *hr* to allow extrusion of the second polar body. The oocytes were then exposed to 1.9 *mM* 6-dimethylaminopurine (6DMAP) prepared in H-SOF for 2 *hr*. The activation process was performed at 39 °*C* in a humidified atmosphere of 5% CO_2_, 5% O_2_, and 90% N_2_.

### In vitro culture

After ICSI, presumptive zygotes were cultured in SOF supplemented with 2% (*v/v*) BME-essential amino acids, 1% (*v/v*) MEM-nonessential amino acids, 1 *mM* glutamine and 8 *mg/ml* fatty acid free BSA at 7% O_2_, 5% CO_2_, and 88% N_2_ at 39 °*C* in humidified air. On days 3 and 5 of culture (Day 0=fertilization), 10% of charcoal stripped fetal bovine serum (FBS) was added to the medium. The culture continued until 8 d post fertilization. The cleavage and blastocyst rates were recorded on days 3 and 7, respectively.

### Assessment of oocyte activation and male pronuclear formation

Sixteen hours after ICSI, the injected oocytes were transferred to ice-cold ethanol containing 10 *mg/ml* Hoechst 33342 for 15 *min*. The oocytes after mounting on glass slide were examined under an epifluorescent microscope (IX71 Olympus, Tokyo, Japan). The criterion used for defining oocyte activation was the presence of at least one pronucleus. The presence of two pronuclei in the absence of sperm head within the oocyte or perivitelline space was an indication of the presence of both female and male pronuclei.

### Statistical analysis

Data were collected over at least 3 replicates. All proportional data were subjected to an arc-sine transformation, and the transformed values were analyzed using one-way ANOVA. When ANOVA revealed a significant effect, the treatments were compared by Fisher LSD method. When an equal variance test failed, treatments were compared by Student-Newman-Keuls Method. When the normality test failed, the Kruskal-Wallis One Way Analysis of Variance on Ranks was applied. Chi-square and Fisher Exact Test were applied when qualitative evaluation was considered. All analyses were conducted with SPSS Version 11.5 (SPPS Inc., Chicago, IL, USA) and p<0.05 was considered significant.

## Results

### Assessment of sperm head decondensation and acrosome reaction in vitro

**Figure 1.**
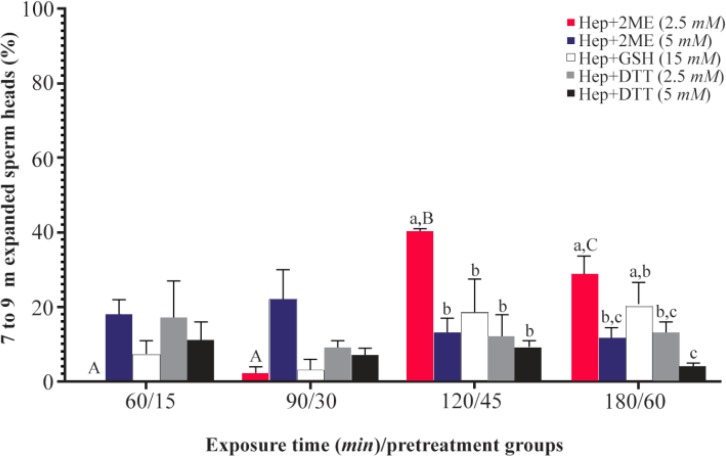
Sperm head expansion in the presence of different reducing agents at different incubation times. Incubation times of 15, 30, 45, and 60 *min* are related to Hep+DTT and 60, 90, 120, and 180 *min* are related to Hep+2ME and Hep+GSH groups. Hep, heparin; 2ME, 2-mercaptoethanol; GSH, glutathione; DTT, dithiotheritol. A–C) Columns with different uppercase letters indicate significant difference between different incubation times in the same treatment group (p<0.001). A–C) Columns with different lowercase letters indicate significant difference between different treatment groups at the same incubation time (p<0.05).

**Figure 2.**
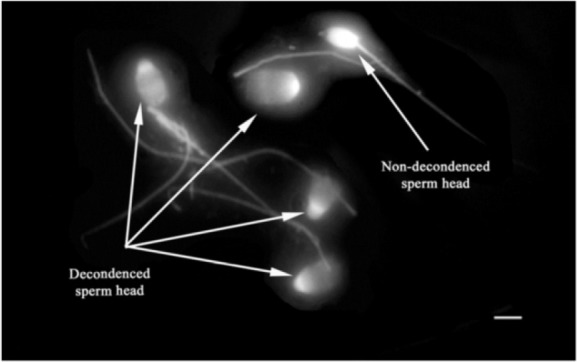
Sperm head decondensation following pretreatment with reducing agents (Bar=5 *μm*).

The greatest percentage of sperm with desired head expansion (7 to 9 *μm*) was achieved in Hep+2ME (2.5 *mM*) group after 2 *hr* of incubation, while at the same time the lowest percentage was observed in Hep+DTT (5 *mM*) group (Figure 1). Similarly, after 3 *hr* of exposure, the highest and lowest percentages of sperm with 7 to 9 *μm* head expansion were observed in the same groups (Figure 2). As shown except for Hep+2ME (2.5 *mM*) group, no significant difference was observed in other groups after different incubation times (Figure 1). Indeed, the positive effect of Hep+2ME on expansion of sperm head was observed only at 2.5 *mM* concentration and after 2 *hr* of incubation. This effect, however, was also evident after 3 *hr* of incubation albeit to a lesser extent. Regarding the induction of acrosome reaction, based on gelatinolysis test, more than 80% of pretreated sperm with ionomycin were acrosome reacted.

### Sperm DNA integrity following in vitro induction of sperm head decondensation

**Figure 3.**
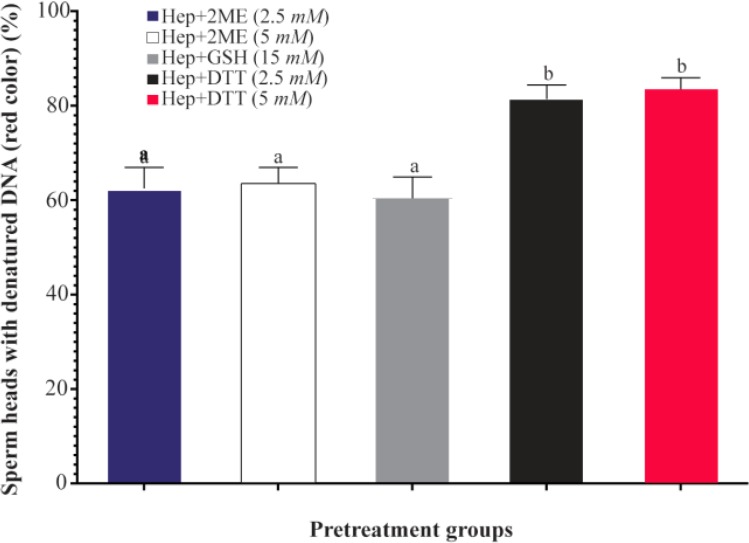
Sperm pretreatment with different disulfide reducing agents on DNA damage assessed by acridine orange. Hep, heparin; 2ME, 2-mercaptoethanol; GSH, glutathione; DTT, dithiotheritol a–b) Columns with different lowercase letters differ significantly (p<0.001).

**Figure 4.**
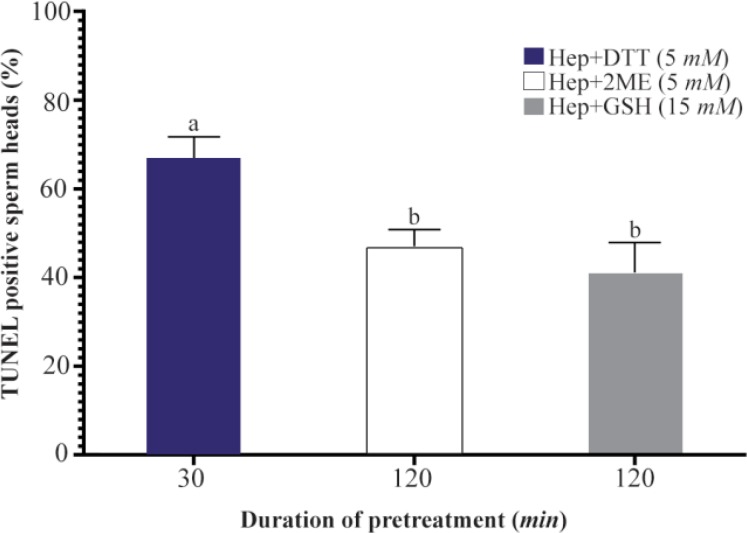
Effects of sperm pretreatment with disulfide bond reducing agents on DNA fragmentation assessed by TUNEL assay. Hep, heparin; 2ME, 2-mercaptoethanol; GSH, glutathione; DTT, dithiotheritol. a–b) Columns not sharing common letters differ significantly (p< 0.05).

As shown (Figure 3), the proportion of sperm with denatured DNA after AO staining (red colored sperm head) in Hep+DTT groups was higher than the other groups (p<0.001). Similarly, the percentage of sperm with fragmented DNA (Tunel positive) in those pretreated with DTT for 30 *min* was greater (p<0.05) than those exposed to 2ME and GSH for 2 *hr* (Figure 4).

### Oocyte activation and male pronuclear formation after intracytoplasmic injection of pretreated sperm

The proportion of activated oocytes was significantly greater in Hep+GSH (92.2%) compared to other groups except for the control and Hep+2ME+injSE groups (Table 1). Regarding male pronuclear formation, the Hep+GSH+coGSH had a highest percentage (61.9%) which was significantly higher than other groups except for the Hep+2ME+coGSH group (Table 1).

**Table 1.** Male pronuclear formation and oocyte activation following ICSI in ovine oocytes injected with pretreated sperm

**Experimental group**	**Oocyte n**	**Non-activated oocyte n**	**Activated oocyte n (%)**		

			**1 Pronucleus**	**2 Pronucleus**	**Total**
**Control**	61	13	32	16(26.2) ^a^	48(78.7) ^a,b,c^
**Hep+ GSH**	64	5	40	19(29.7) ^a,c^	59(92.2) ^a,c^
**Hep+GSH+co.GSH**	84	18	14	52(61.9) ^b^	66(78.6) ^b^
**Hep+GSH+inj.SE**	55	13	28	14(25.4) ^a^	42(76.4) ^b^
**Hep+ 2ME**	79	8	42	28(35.4) ^a,c^	70(88.6) ^c^
**Hep+2ME+co.GSH**	60	16	17	27(45) ^b,c^	44(73.3) ^b^
**Hep+ 2ME+inj.SE**	41	8	24	9(21.9) ^a^	33(80.5) ^a,b,c^
**AR-Hep+2ME+co.GSH**	47	16	15	16(34) ^a,c^	31(65.9) ^b^
**AR-Hep+GSH+co.GSH**	58	18	23	17(29.3) ^a,c^	40(69) ^b^

a–c) Numbers with different lowercase superscript letters in the same column differ significantly (p<0.05). Hep, Heparin; GSH, Glutathione; co.GSH, coinjection with glutathione; inj.SE, coinjection with sperm extract; 2ME, 2-Mercaptoethanol; AR, Acrosome reacted.

### Developmental competence of oocytes injected with pre-treated sperm

No significant difference was observed in cleavage and blastocyst rates between groups though the blastocyst rate was insignificantly higher in Hep+2ME+ coGSH (21±7.4) compared to other groups (Table 2).

**Table 2.** Developmental competence of ovine ICSI oocytes injected with pretreated sperm with different protocols

**Experimental groups**	**Oocyte n**	**Cleavage n (Mean±SEM)**	**Blastocyst n (Mean±SEM)**
**Control**	155	125 (81.9±1.9)	22 (17.4±1.8)
**Hep+GSH+co.GSH**	105	89 (84.4±7.9)	16 (16.6±2.9)
**Hep+2ME+co.GSH**	74	64 (85.9±2)	15 (21±7.4)
**AR+Hep+2ME+co.GSH**	52	47 (91±6.3)	9 (19.2±3.6)

Hep, Heparin; GSH, Glutathione; co.GSH, coinjection with glutathione; 2ME, 2-Mercaptoethanol; AR, Acrosome reacted.

## Discussion

The inadequate sperm chromatin decondensation and lack of MPN formation together with impaired oocyte activation are the major causes behind the poor ICSI efficiency ^4,5^.

Theoretically, a possible reason for this defect may be attributed to the inability of Phospholipase C zeta (PLCζ), a Sperm-borne Oocyte-activating Factor (SOAF), to penetrate through the sperm plasma membrane, resulting in a limited amount of PLCζ available for oocyte activation ^28,36^.

Apart from sperm factors in induction of oocyte activation, the oocyte contents, especially glutathione, seem to be the requisites for disulfide bonds reduction in the sperm nucleus and promotion of male pronuclear formation during fertilization ^6,37^.

To improve MPN formation following ICSI, sperms have often been pre-treated by various methods ^5,6,16, 25,38^

These treatments focused on the strategy that reduction of disulfide bonds and removal of sperm membranes, through the easier access of oocyte cytoplasm to SOAF, may improve male pronuclear formation and the subsequent embryo development.

It was previously reported that in ovine species, an injected sperm itself poorly stimulates the activation process in ewe oocytes and that the additional chemical activation is the requisite for development of ewe ICSI oocytes to the blastocyst stage. The majority of resulting embryos, however, were parthengenic, due to lack of MPN formation and failure of its contribution at fertilization ^12^.

In the first experiment of the current study among different disulfide bonds reducing agents, the combination of Hep+2ME was more efficient in sperm head decondensation, 7 to 9 *μm*, *in vitro* compared to other groups (Figure 2). This effect, however, was evident after 120 *min* of incubation at the presence of 2.5 *mM* 2ME compared to other incubation times and even higher 2EM concentration (Figure 1).

Considering the effects of different reducing agents on sperm chromatin integrity, the combination of Hep+ DTT at both concentrations of 2.5 and 5 *mM* DTT had the most detrimental effect. Indeed, the percentages of sperm with denatured (AO positive) and fragmented DNA (Tunel positive) in Hep+DTT group were significantly higher than Hep+GSH and Hep+2ME groups (Figures 3 and 4). Apart from DNA damage, the percentage of sperm with head deformity (bent head) was higher in Hep+DTT group compared to other groups (unpublished data) which were in agreement to our previous report ^12^.

The adverse effect of DTT on integrity of sperm chromatin has previously been reported in other species. In pig, the paternal chromosomal integrity of sperm treated with DTT was compromised compared to GSH treated sperm. Additionally, while the majority of blastocysts derived from control and GSH-treated sperm were diploid, the blastocysts derived from DTT-treated spermatozoa were haploid ^39^. There is also evidence indicating that while the expression of pluripotent and anti-apoptosis markers in blastocysts derived from sperm pretreated with heparin-GSH were comparable to IVF, their expression in blastocysts derived from DTT pretreated sperm was compromised ^40^.

In our second experiment, the injection of sperm with decondensed head, 7 to 9 *μm*, pretreated with either GSH+Hep or 2ME+Hep could increase, though insignificantly, the percentage of activated oocytes compared to control. In both groups, co injection of either GSH or SE with pretreated sperm not only failed to increase the proportion of activated oocytes but also decreased the related percentage in some groups (Table 1). It is unclear why coinjection of GSH or SE with pretreated sperm in some groups decreased the proportion of activated ICSI oocytes.

Whether the injection volume was inadvertently increased in case of co-injection and/or whether SE had some deleterious components, *e.g*. proteases, which finally could adversely affect the number of activated oocytes, further investigations should be carried out. The MPN formation, however, in GSH+Hep and 2ME+Hep groups when sperm coinjected with GSH was greater than other groups. Indeed, none of the other treatment approaches including coinjection of SE and injection of acrosome reacted sperm could increase MPN formation. As known, GSH is the requisite to ensure sperm chromatin decondensation ^6^ so that GSH synthesis during oocyte maturation is an important factor for promoting the ability of oocytes to form a MPN ^41,42^. In our study condition, while co injection of GSH could increase the rate of MPN, co injection of SE with pretreated sperm had no positive effect on MPN formation compared to control (Table 1). One possibility for ineffectiveness of SE co injection might be related to probable destruction of SOAF in the medium. In mouse, there is evidence indicating the instability of SOAF activity in SE at 37 °*C* ^35^. Additionally, whether how the extent of time interval between sperm SE preparation and its co injection could deteriorate the effectiveness of SE on MPN formation might be other possibility for ineffectiveness of SE co injection.

The other point in our study was the lack of direct association between MPN formation and oocyte activation that suggests the activation of ovine oocytes is not necessarily associated with MPN formation ^43,44^. Indeed, it seems MPN formation in sheep needs further requirements. As shown (Table 1), there was no difference in proportions of activated oocytes between treated and control groups and it seems the post-ICSI treatment of oocytes with ionomycin and 6DMAP was enough for oocyte activation. Indeed, sperm pretreatment and/or GSH and SE co-injections had no additive effects on oocyte activation. Whereas the higher MPN formation was observed in Hep+GSH+ co.GSH and Hep+2ME+co. GSH groups, it can be assumed that the higher MPN formation in these groups may be related to the GSH co-injection.

In the third experiment, the effect of acrosome removal before injection of pretreated sperm on MPN formation was investigated. The toxic effects of the acrosome and its contents such as deformation of the oocyte with an irregular appearance due to morphological and ultrastructural alterations, edema or cytolysis, and oocyte cytoskeleton disruption have been described by Morozumi & Yanagimachi *et al* ^23^. On the other hand, there are many reports indicating that the induction of sperm acrosome reaction from several species prior to ICSI can accelerate the oocyte activation and fertilization rate ^13,23,45^. In contrast to what stated above, in our study condition, induction of acrosome reaction prior to ICSI had no positive effect on oocyte activation or MPN formation. In this context, there is evidence that indicates exogenous induction of the acrosome reaction is not necessary for MPN formation and activation of porcine oocytes ^2^.

In the last part of our study, to evaluate the effect of sperm pretreatment on the subsequent embryo development following ICSI, three groups of pretreated sperm were applied (Hep+GSH+co.GSH, Hep+2ME+ co.GSH, and AR-Hep+2ME+co.GSH; Table 1) based on the results of our previous experiments. As shown (Table 2), no significant difference was observed in cleavage and blastocyst rates between groups with the lowest and highest blastocyst rates in Hep+GSH+ co.GSH and Hep+2ME+co.GSH groups, respectively. Indeed, despite our expectation, no direct relationship was observed between MPN formation and oocyte activation with embryo development.

## Conclusion

In conclusion, in our study condition, despite the insignificant difference in embryo development between groups injected with pretreated sperm or even in those receiving further oocyte activation compared to control, the higher MPN formation in some treated groups indicated the greater chance of normal fertilization in those groups compared to control.
